# Environmental Factors Modulate the Role of *orf21* Sigma Factor in Clavulanic Acid Production in *Streptomyces Clavuligerus* ATCC27064

**DOI:** 10.3390/bioengineering9020078

**Published:** 2022-02-16

**Authors:** Luisa F. Patiño, Vanessa Aguirre-Hoyos, Laura I. Pinilla, León F. Toro, Rigoberto Ríos-Estepa

**Affiliations:** Grupo de Bioprocesos, Departamento de Ingeniería Química, Universidad de Antioquia UdeA, Calle 70 No. 52–21, Medellín 050010, Colombia; luisa.patinoc@udea.edu.co (L.F.P.); vanessa.aguirre@udea.edu.co (V.A.-H.); laura.pinilla@udea.edu.co (L.I.P.); lfelipe.toro@udea.edu.co (L.F.T.)

**Keywords:** *Streptomyces clavuligerus*, clavulanic acid, sigma factors, gene expression

## Abstract

Sigma factors and sigma factor-related mechanisms control antibiotic production in *Streptomyces*. In this contribution, the *orf21* gene was overexpressed in the wild-type strain of *Streptomyces clavuligerus* ATCC2764, yielding *S. clavuligerus*/pIORF21, to further evaluate its regulatory effect on clavulanic acid (CA) biosynthesis under different culture medium conditions. The *orf21* overexpression, regulated under the constitutive promoter ermE*, led to 2.6-fold increase in CA production in GSPG medium, and a 1.8-fold decrease using ISP medium. As for GYM and MYM media, *S. clavuligerus*/pIORF21 strain showed higher aerial mycelium production compared to control. Glycerol uptake rate profile was affected by *orf21* overexpression. Furthermore, in GSPG, *S. clavuligerus*/pIORF21 slightly increased the expression of *adpA* and *gcas* genes, whilst, in ISP, the *claR* gene expression was drastically reduced, which is consistent with a decreased CA production, observed in this medium. These findings suggest the protein encoded by the *orf21* gene plays a role in the regulation of CA biosynthesis as a response to the nutritional composition of the medium.

## 1. Introduction

Clavulanic acid (CA) is an irreversible inhibitor of classes A and D serine β-lactamases [1]. The use of β-lactam antibiotics in combination with CA is a common treatment against infections caused by organisms suspected to be β-lactamase producers [2]. This combination was registered in the World Health Organization (WHO) list of essential medicines (2019) [3]. CA is produced in submerged cultures using the filamentous Gram-positive bacterium *Streptomyces clavuligerus* [4]; its production cost is relatively high mainly due to the low concentration levels, achieved during fermentation, and uncontrolled degradability [5]. Due to the clinical importance of CA as a drug for the treatment of antimicrobial resistance, significant progress has been made to improve CA production, e.g., through the study of the effect of environmental parameters on cell culture, culture media composition, and genetic rearrangements to enhance metabolic pathway fluxes involved in its biosynthesis [5,6].

**Figure 1:**
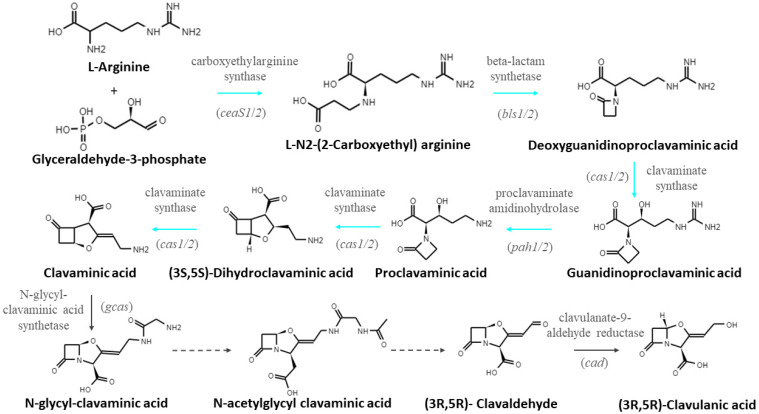
The CA biosynthetic pathway in *S. clavuligerus*. Solid lines represent known steps and dashed lines indicate unknown parts of the pathway. Cyan lines represent early steps and gray lines the late steps.

CA biosynthesis begins at the L-arginine and glyceraldehyde-3-phosphate condensation, catalyzed by carboxyethylarginine synthase (CeaS); the next five consecutive enzymatic reactions, generally referred to as early steps, lead to (3S,5S)-clavaminic acid formation (Figure 1) [2]. The formation of clavaminic acid is an important branch point in the CA pathway since it can either be converted to (3R,5R)-clavulanic acid, or to 3S,5S clavams, by the late reactions of the pathway [7]. For CA biosynthesis, different compounds result from the intermediate steps involved in the transition of the clavaminic acid into CA. The first late reaction is the conversion of clavaminic acid to N-glycyl-clavaminic acid, catalyzed by N-glycyl-clavaminic acid synthase (Gcas); then, a possible acetylation of N-glycyl-clavaminic acid leads to the N-acetylglycyl-clavaminic acid molecule, which binds to the OppA2 protein to be transported to the place in the cell where the next enzymatic reactions occur [8]. Subsequently, for clavaldehyde biosynthesis, the N-acetylglycyl group from N-acetylglycyl-clavaminic acid must be released through up-to-date unknown enzymatic steps. The final step of the pathway is the conversion of clavaldehyde to CA by clavaldehyde reductase (Cad) (Figure 1) [8]. Although most of the genes involved in the CA metabolic pathway are well known, the regulation of CA synthesis is complex and involves cluster-situated regulators, global mechanisms, and signaling cascades [1].

The biosynthetic genes responsible for CA production are regulated by pathway-specific transcriptional regulators. For instance, the *claR* gene is a transcriptional activator located within the CA gene cluster, which upregulates the ‘late’ biosynthetic genes involved in the conversion of clavaminic acid to CA [2]. Alternatively, *ccaR* is located within the adjacent cephamycin C gene cluster and encodes the CcaR protein which positively regulates both cephamycin C and CA production [9]. Transcriptional analysis suggests that CcaR modulates CA production directly, by regulating the expression of some ‘early’ genes, and indirectly, by regulating the expression of *claR* [2].

Furthermore, expression of transcriptional regulators is controlled by global regulatory mechanisms, bound to the production of antibiotics, which depend on environmental conditions and the bacterial physiological state, e.g., the AdpA protein. AdpA affects aerial mycelium formation in *S. clavuligerus* and positively modulates the production of CA in transformants carrying multiple *adpA* copies [10]. Similarly, environmental signals trigger a cellular response to stress generated by nutrient starvation and adverse growth conditions, among others. Pinilla et al. (2019) observed that CA synthesis in *S. clavuligerus* occurs under stress conditions induced by exhaustion of amino-acid content [11]. Under this depletion, the overexpression of the metalloprotease SCLAV_4359, at the onset of cell lysis, was observed, suggesting that the synthesis of this protease may be directly involved in morphological differentiation and indirectly towards CA production [11].

Sigma factors and sigma factor-related mechanisms also control antibiotic production and concomitantly other functions of *Streptomyces* primary metabolism [2]. Jensen et al. (2008) identified the open reading frame denoted as *orf21*, which was annotated as a putative sigma factor, located downstream of the CA gene cluster in *S. clavuligerus* ATCC 27064 and whose interruption did not affect CA production in a glycerol-arginine (GA) medium [12]. Subsequently, Jnawali et al. (2010) found that *orf21* overexpression in a multicopy plasmid increased CA production by 1.43-fold during cultivation in SA medium [13]. Likewise, *orf21* was disrupted and its mutation in *S. clavuligerus* resulted in a 10–15% decrease in CA production in the same cultivation medium [13]. Although *orf21* has been previously studied, its physiological role in *S. clavuligerus* and its relationship with CA biosynthesis is still matter of discussion since the specificity of the ORF21 effect on CA production is variable across different laboratories and seems to be closely linked to operational differences during cultivation, e.g., medium composition [12,13].

In this contribution, we report on the overexpression of *orf21* in *S. clavuligerus* ATCC 27064 and its potential regulatory effect on CA synthesis and other cell physiological processes in a nutrient-deficient environment, unfavorable for CA biosynthesis, and under favorable nutrient conditions for CA production. This research also seeks to further contribute to a better understanding of the complex regulatory network that characterizes secondary metabolite biosynthesis in *S. clavuligerus* ATCC 27064.

## 2. Materials and Methods

### 2.1. Bacterial Strains, Plasmids, and Culture Conditions

Microorganisms and plasmid vectors, used in this study, are listed in Supplementary Table S1 *Escherichia coli* strains were grown in either Luria broth (LB) (Merck) or agar plates at 37 °C, supplemented with antibiotics, when necessary (ampicillin 100 μg/mL, apramycin 50 μg/mL). DNA handling was carried out using *E. coli* DH5α, and then *E. coli* DSM 11539 to avoid the *S. clavuligerus* ATCC 27064 restriction barrier, prior to running the protoplast isolation protocol. *S. clavuligerus* wild-type and mutant strains were grown in trypticase soy broth (TSB) at 28 °C and 220 rpm; the mycelial culture was stored at −80 °C in a glycerol solution (20% *v*/*v*). The morphological differentiation analyses were carried out in MYM and GYM plates [14]. The GYM medium composition was (mg/mL): glucose, 4; yeast extract, 4; malt extract, 10; CaCO_3_, 2; and agar, 12. Apramycin (40 μg/mL) was added to *S. clavuligerus* mutant strain cultures as needed.

For protoplast transformation, *S. clavuligerus* was cultured in the YEMEG medium containing (g/L): yeast extract, 3; malt extract, 3; peptone, 5; and sucrose, 150; supplemented with 0.2% MgCl_2_·6H_2_O (2.5 M) and 2.5% glycine (20%), at pH 7.0, 28 °C, and 220 rpm to get an OD600 value between 7 and 10. As for protoplast regeneration, the R2YEG medium was used and its composition was as follows (g/L): sucrose, 103; K_2_SO_4_, 0.25; MgCl_2_ 6H_2_O, 10.12; glycerol, 10; yeast extract, 5; casamino acid, 0.1; agar, 22; and trace element solution, 2 mL. The composition of the trace element solution was as follows (mg/L): ZnCl_2_, 40; FeCl_3_·6H_2_O, 200; CuCl_2_·2H_2_O, 10; Na_2_B_4_O_7_·10H_2_O, 10; (NH_4_)_6_Mo_7_O_24_·4H_2_O, 10. The R2YEG medium was supplemented with KH_2_PO_4_ (0.5%), 1 mL; CaCl_2_ (0.25 M), 8 mL; L-proline (20%), 1.5 mL; TES (5.73% pH 7.2), 10 mL; NaOH (1 M), 0.5 mL.

A seed medium was used for *S. clavuligerus* cultivation at pH 6.8; its composition was as follows (g/L): glycerol, 15; peptone, 10; malt extract, 1.0; MgSO_4_, 0.75; MnCl_2_, 0.0001; FeSO_4_, 0.001; ZnSO_4_ 0.001; MOPS, 21; K_2_HPO_4_, 0.8. For CA production the following culture media were used. GSPG medium (g/L): glycerol, 15; sucrose, 20; proline, 2.5; glutamic acid, 1.5; NaCl, 5; K_2_HPO_4_, 2; CaCl_2_, 0.4; MnCl_2_·4H_2_O, 0.1; FeCl_3_·6H_2_O, 0.005; ZnCl_2_, 0.005; MgSO_4_·7H_2_O, 1; pH 7.0 [15]. GSPG was used as pre-culture and culture medium. Soy protein isolate (ISP) medium (g/L): glycerol, 30; soy protein isolate, 25; K_2_HPO_4_, 0.8; MgSO_4_·7H_2_O, 0.75; MnCl_2_·4H_2_O, 0.0001; FeSO_4_·7H_2_O, 0.001; ZnSO_4_·7H_2_O, 0.001; MOPS, 21.0; and pH fixed at 6.8 [5]. The pre-culture medium had the same composition as that of the culture medium, except for glycerol concentration which was adjusted to 15 g/L. All *S. clavuligerus* cultures were carried out in 250-baffled Erlenmeyer flasks containing 50 mL of medium. Pre-culture flasks were inoculated with seed medium (10% *v*/*v*), and the cultivation medium was inoculated with 10% *v*/*v* of pre-culture medium. Cultures were incubated for 120 h, at 220 rpm and 28 °C.

### 2.2. Construction of Recombinant Plasmids

Chromosomal DNA of *S. clavuligerus* ATCC 27064 was isolated using a modified salting out procedure as described elsewhere [15]. *S. clavuligerus* ATCC 27064 DNA was used to amplify the *orf21* gene by PCR. Primers designed for PCR amplification were synthesized by Macrogen, Korea (See supplementary Table S2). PCR mix was prepared as follows: 5 µL 10X PCR buffer, 1.5 µL 10 mM dNTPs solution and 2.5 μL DMSO, 1.2 μL for each primer (10 µM), 1 μL template DNA (50 ng), 0.5 μL of 1.25 U Taq DNA polymerase (New England Biolabs), and 37.1 μL of dH_2_O.

The PCR cycling conditions started with an initial denaturation step (1 min at 95 °C), followed by 30 cycles of amplification (30 s at 95 °C for denaturation, 30 s at Tm −5 °C for annealing, 2 min/1 kb at 72 °C for extension), and a final extension step at 72 °C for 5–20 min. Enzymes used in PCR and cloning experiments were acquired from New England Biolabs, Ipswich, MA. Colony PCR amplification was performed as described by Sambrook [15], under slight modifications for *S. clavuligerus* reaction adjustment. Samples taken from plated colonies were re-suspended in 50 μL dH_2_O and lysed at 95 °C for 30 min. Then, 5 μL of cell lysate were used as template for the previously described standard PCR reaction mix.

### 2.3. Construction of Recombinant Plasmids and Transformation

The *orf21* gene was subcloned in the pTZ57R vector (InsTAclone PCR Cloning Kit, Thermo) and then inserted into pIB139 [16] via *Xba*I/*Nde*I restriction sites to yield pIORF21. Construction was verified by PCR and sequencing. The plasmids (pIORF21 and pIB139) were propagated in *E. coli* DH5α cells and subsequently introduced into the *E. coli* DSM 11539 host.

The constructed plasmids were introduced into *S. clavuligerus* by a modified polyethylene glycol (PEG)-mediated protoplast transformation procedure [17]. The protoplasts were obtained from cultures grown at 30 °C in YEMEG medium under previously described conditions. The *S. clavuligerus* mycelium was collected and washed twice in a 10.3% sucrose solution, as for protoplasting [17]; the pellet was suspended in 2 mL of P buffer, containing 50 μL of lysozyme (20 mg/mL) and incubated at 30 °C for 20 min.

After dilution of the lysis solution with 8 mL of P buffer, the protoplast suspension was centrifuged twice and suspended in 0.8 mL of P buffer. The protoplast suspension was incubated at 42 °C for 10 min. Following, 1 μg of plasmid DNA was added to 100 μL of protoplast solution and mixed with 0.2 mL of PEG 1000 (40%). After 1 min at room temperature, the solution was further diluted by adding 1 mL of P buffer. The protoplasts were collected by centrifugation at 4000 rpm and suspended in 1 mL of P buffer. Then, the protoplasts were plated (0.1 mL per plate) in R2YEG media (previously dehydrated in a laminar flow cabinet for 2 h) and incubated at 28 °C. About 36 h later, when small colonies were observed, 2 mL aliquots of sterile dH_2_O containing 40μg/mL of apramycin were spread onto plates, and incubation further continued for 3 days. The recombinant strains were termed as *S. clavuligerus*/pIORF21 and *S. clavuligerus*/pIB139, respectively.

The presence of the plasmid pIORF21 in *S. clavuligerus* was confirmed by PCR using the cloned gene reverse primer and a forward primer, designed from an inner region of the promoter ermE*, present in the plasmid sequence. To confirm insertion of pIB139 in *S. clavuligerus* the primers were designed from the apramycin resistance gene, *aac(3)IV* (Supplementary Table S2). The control strain (*S. clavuligerus*/pIB139 having vectors without any gene inserted) was constructed to determine the potential effect of pIB139 on CA yield for a recombinant strain and to evaluate the effect of the cloned gene on CA production without considering effects caused by the presence of pIB139.

### 2.4. Analytical Techniques

The analyses of *S. clavuligerus* and its recombinants, including control cultures, were performed by triplicate; experimental data were calculated as the mean value with the error indicated by the standard deviation. For sampling, 2 mL aliquots were taken from each flask every 24 h during the fermentation process (120 h). For CA quantification, the culture samples were centrifuged at 14,000 *g* for 10 min at 4 °C and filtered (0.22 µm). CA quantification was carried out as follows: culture samples were centrifuged at 14,000 *g* for 10 min at 4 °C and filtered (0.22 µm). CA was determined by HPLC Agilent 1200 (Agilent Technologies, Waldbrom, Germany) equipped with a Diode Array Detector (Agilent Technologies, Palo Alto, CA, USA) at 312 nm, using a reverse phase ZORBAX Eclipse XDB-C18 (4.6 mm × 150 mm, 18μm Agilent Technologies, Palo Alto, CA, USA) column; 94% *v*/*v* KH_2_PO_4_ (50 mM, pH 3,2) and a 6% *v*/*v* methanol solution was used as mobile phase at 1 mL/min. CA was imidazole-derivatized at a ratio 1:3; the reaction was kept at 28 °C for 15 min [18].

Glycerol concentration was determined by HPLC-RID [19]. Biomass growth was determined using the dry weight technique; before measuring (gDWC l-1), cells were washed twice with deionized water and dried at 80 °C for 24 h.

### 2.5. Statistical Analysis

The experimental results were statistically analyzed using the Statgraphics^®^ software (version 18). For biomass, glycerol, and CA results, data were evaluated by the Shapiro–Wilk normality test. Parametric data were subjected to analysis of variance (ANOVA) and Tukey’s HSD test. Significance level was set as *p* < 0.05.

### 2.6. RNA Extraction and RT-qPCR Analysis

Total RNA was extracted from mutants and wild-type *S. clavuligerus* cell cultures at 72 and 96 h of cultivation, when CA concentration was the highest of the tested conditions. Samples were centrifuged at 10,000 *g* for 15 min; cell pellets were immediately stored at −80 °C. Total RNA was isolated using Trizol^®^ (Sigma^®^), following the manufacturer’s instructions [20]. All RNA preparations were treated with RNase-free DNase I (Promega^®^) to eliminate genomic DNA contamination; RNA purity and concentration was determined using QIAEXPERT^®^ equipment (QIAGEN^®^). Gene expression analysis and quantification of some CA biosynthesis-related genes, were assessed from two samples coming out of two different culture media (GSPG and ISP), using RT-qPCR as described by R. Álvarez-Álvarez et al. [19]; assays involved the $2^{\left(-{\Delta \Delta C}_{t}\right)}$ method [21] and the constitutive housekeeping *hrdB* gene as a control for replication threshold [22]. Data for relative gene expression were normalized using the log 2 $[2^{\left(-{\Delta \Delta C}_{t}\right)}].$ The cDNAs were synthesized as described by R. Álvarez-Álvarez et al. [19]. Negative controls were used to determine DNA contamination. As for the transcriptional analysis using RT-qPCR, the following genes were tested: *ccaR*, *adpA*, *claR*, *gcas*, *orf21*, and SCLAV_4359. All primers used in the experiment are shown in Supplementary Table S2. Equal RNA quantity (1 µg) was used for all RT-qPCR experiments.

### 2.7. Phylogenetic Analysis and Prediction of DNA Motifs Bound

Phylogenetic inferences were performed using ORF21 homologous proteins and different extracytoplasmic function (ECF) sigma factors. ORF21 homologous proteins were mined using the NCBI protein BLAST algorithm (www.ncbi.nlm.nih.gov/BLAST/ accessed on 6 November 2021) with a PAM 70 as substitution matrix. RefSeq protein sequences were chosen according to its identity percentage (≥50%) and adequate *E*-value confidence criterion (≤1×10^−37^). Following, a Bayesian tree was constructed using MrBayes (MB) V3.2 [23] setting the Jones Gamma distributed amino acid substitution model, with 1,000,000 generations sampled every 1000 generations and keeping the other parameters as default values. The convergence of the Markov–Monte Carlo interactions was assessed with the potential scale reduction factor (PSFR = 1) [24] and the standard deviation of split frequencies (0.008).

The ECF sigma factor sequences from different *Streptomyces* species were selected from GenBank. The phylogenetic tree for ECF sigma factors was inferred using the Maximum Likelihood method [25] and JTT matrix based-model [26]. The bootstrap consensus tree inferred from 1000 replicates was taken to represent the evolutionary history of the taxa analysis. The rates among sites were treated as a Gamma distribution using 4 Gamma Categories (Gamma Distribution option). This analysis involved 30 amino acid sequences. Evolutionary analyses were conducted in MEGA11 [27].

Six putative regulatory sequences were compiled from the 250 nucleotide-long sequences located upstream of the *claR* gene of *S. clavuligerus*, *S. jumonjinensis*, and *S. katsurahamanus*, and upstream of the *adpA*, *ccaR*, and *gcas* genes of *S. clavuligerus*. The sequences were analyzed in MEME SUITE 5.1.1 [28], while motifs found were analyzed using TOMTOM [29] for further comparisons. The highest-scoring motif was selected from all generated motifs.

## 3. Results

### 3.1. Morphology, Biomass Growth, and CA Production

**Figure 2:**
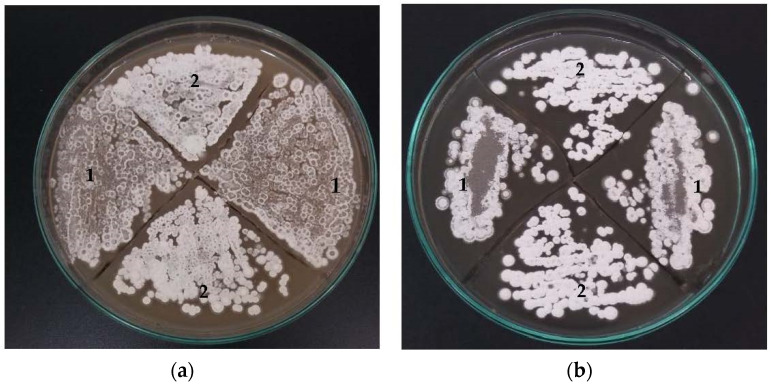
Aerial mycelium formation in solid medium with apramycin 40 µg/mL. (1) *S. clavuligerus*/pIB139. (2) *S. clavuligerus*/pIORF21. (**a**) After 5 days of growth in GYM. (**b**) After 8 days of growth in MYM. In both culture media, *S. clavuligerus*/pIORF21 stimulated aerial mycelium formation, compared to control.

Since *orf21* transcription is activated by unknown regulatory mechanisms involving diverse environmental cues, in this contribution we assessed the effect of overexpressing *orf21* into pIB139 under ermE* promoter to increase its transcription rate and to search for its regulatory role in CA biosynthesis under different nutritional conditions. Figure 2 shows the results for aerial mycelium formation of *S. clavuligerus*/pIORF21, in GYM and MYM media. *S. clavuligerus*/pIORF21 produced aerial mycelium after 5–8 days of cultivation; only sparse mycelium was observed in the control strain (*S. clavuligerus*/pIB139) (Figure 2).

**Figure 3:**
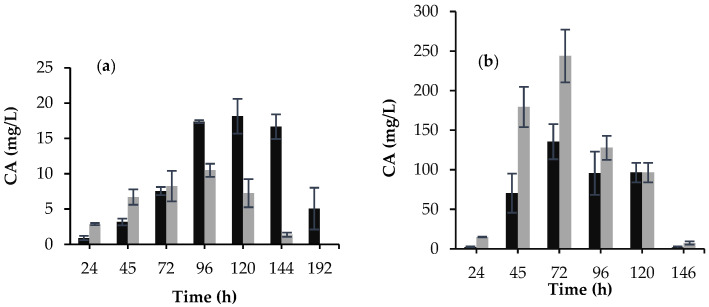
Evaluation of CA production under different nutritional conditions. (**a**) GSPG medium. (**b**) ISP medium. In black *S. clavuligerus*/pIORF21; in dark gray *S. clavuligerus*/pIB139. Each value corresponds to the mean of three flask replicates (*n* = 3). Error bars indicate standard deviation values.

*S. clavuligerus*/pIORF21 and the control strain were evaluated for CA production in two different culture media. For GSPG, the most significant increase in CA concentration was observed in *S. clavuligerus*/pIORF21 after 96 h (17.4 mg/L) and 120 h (18.1 mg/L) of cultivation, representing 1.7 and 2.6-fold (*p* < 0.05) increase, respectively. CA production for the control strain, was 10 and 7 mg/L, at 96 and 120 h, respectively (see Figure 3a). Surprisingly, CA production was maintained for *S. clavuligerus*/pIORF21 until 144 h of cultivation, in contrast to the control strain for which a continuous decline in product accumulation was observed, from 96 to 144 h (Figure 3a).

**Figure 4:**
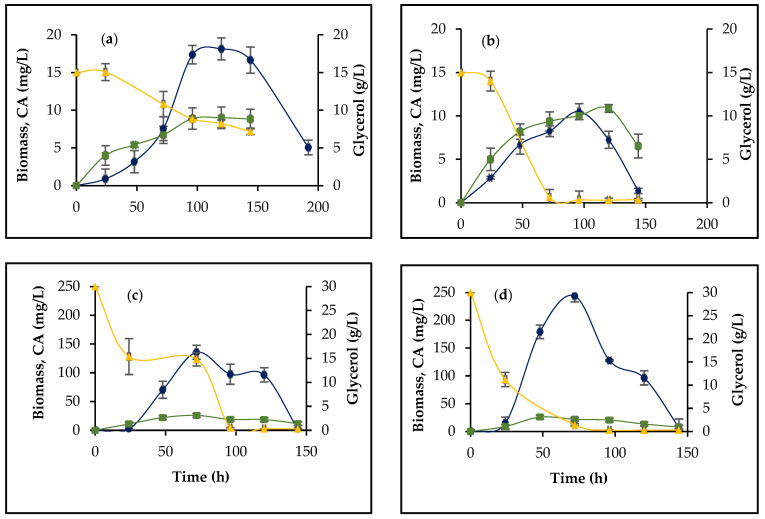
Dynamics of biomass, glycerol, and CA production. (**a**) *S. clavuligerus*/pIORF21 in GSPG. (**b**) *S. clavuligerus*/pIB139 in GSPG. (**c**) *S. clavuligerus*/pIORF21 in ISP. (**d**) *S. clavuligerus*/pIB139 in ISP. Blue—CA; green—biomass; and yellow—glycerol. Each value corresponds to the mean of three flask replicates (*n* = 3). Error bars indicate standard deviation values.

Regarding biomass formation, there was a significant (*p* < 0.05) decrease in biomass growth for *S. clavuligerus*/pIORF21 compared to the control strain, during the exponential growth phase (48 and 72 h), as shown in Figure 4a,b. This reduction, as well as a longer stationary phase, might be related to the kinetics of CA production in *S. clavuligerus*/pIORF21, using GSPG. As for ISP, *S. clavuligerus*/pIORF21 performance, in terms of CA production, was quite different; interestingly, *orf21* overexpression, under the evaluated conditions, showed a low CA production, compared to the control strain (Figure 3b). Concerning biomass growth in ISP, a similar outcome (*p* > 0.05) was observed for *S. clavuligerus*/pIORF21 and *S. clavuligerus*/pIB139 (see Figure 4c,d).

### 3.2. The Effect of orf21 Overexpression on Glycerol Uptake and Ammonium Accumulation

The concentration of the carbon (and/or nitrogen) source performs as a signal for the onset of secondary metabolism. Hence, glycerol uptake, as the main carbon source for *S. clavuligerus*, was measured to evaluate whether *orf21* overexpression affected primary metabolism. The uptake rate of glycerol, shown by *S. clavuligerus*/pIORF21 and the control strain, was significantly different (*p* < 0.05) in both culture media. In *S. clavuligerus*/pIORF21 (Figure 4a,c), glycerol concentration gradually decreased, leading to 7.2 g/L of residual glycerol at 144 h, in GSPG. In contrast, for *S. clavuligerus*/pIB139 (Figure 4b,d), glycerol concentration rapidly decreased until 72 h of cultivation, when it was completely depleted in both culture media. For ISP and GSPG, the specific consumption of substrate in *S. clavuligerus*/pIORF21 decreased notably compared to the control strain (see Table 1). Apparently, *orf21* overexpression played an important role in the metabolism of substrate, independent of the culture medium composition.

Likewise, to connect ammonium accumulation (as a stressful characteristic of GSPG) with CA biosynthesis, its production was compared between GSPG and ISP. A lower ammonium production was observed for ISP compared to GSPG. For each medium, ammonium production did not vary significantly (*p* > 0.05) between strains (Supplementary Figure S1), indicating that *orf21* overexpression has no effect on ammonium accumulation.

### 3.3. Differential Transcription Analysis of Genes Involved in CA Biosynthesis

**Figure 5:**
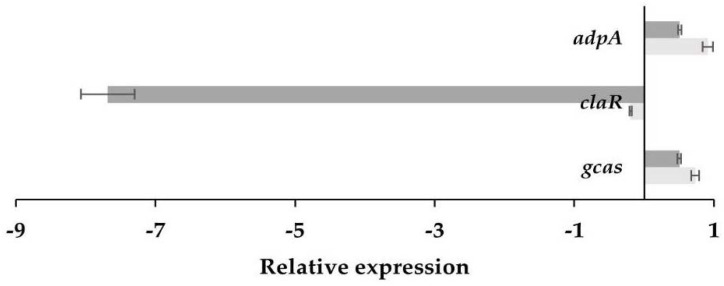
Effects of *orf21* overexpression on the transcription of genes related to CA biosynthesis. RT-qPCR results for *S. clavuligerus*/pIORF21 compared to *S. clavuligerus*/pIB139. In light gray the results for GSPG. In dark gray the results for ISP. The error bars indicate the standard error.

To assess whether some genes associated with CA biosynthesis (*ccaR*, *claR*, *adpA*, SCLAV_4359, *orf21*, and *gcas*) are under the control of *orf21*, a transcription analysis was carried out using RT-qPCR for *S. clavuligerus*/pIORF21 and *S. clavuligerus*/pIB139, grown in two different culture media, (see Figure 5). Transcriptional analysis was performed using, as template, RNA isolated from strains cultured in GSPG medium during 96 h; at this time, CA production started to increase, compared to control (Figure 3a). The gene *gcas* was moderately expressed compared to control; the transcription rate increased by 1.7-fold (Figure 5). Further, *adpA*, a pleiotropic regulator related to the biosynthesis of antibiotics and morphological differentiation in *S. clavuligerus* [10], showed a slight expression increment (1.9-fold). No differences in amplification for *ccaR*, *orf21*, *claR*, and SCLAV-_4359 were observed between the studied strains (data not shown).

RNA samples for transcriptional studies with ISP were harvested at the peak of maximum CA production (72 h) (see Figure 3b). No differences in gene expression were observed, except for *claR*, for which gene expression was greatly reduced (200-fold) (Figure 5). Supplementary Table S3 shows the normalized data for fold change.

### 3.4. Phylogenetic Inferences and DNA-Binding Motifs Prediction

To infer phylogenetic relationships and interplays among ORF21 and other regulators of CA biosynthesis, 60 homologous protein sequences for ORF21 were mined. These proteins belong to microorganisms of the order Actinomycetales, e.g., the genera Nonomuraea, Actinomadura, and Streptomyces. It was found that ORF21 sequence shares homology to SigL RNA polymerase sigma factor (GenBank accession numbers) WP_153520658.1 and WP_153484481.1 for *S. jumonjinensis* and *S. katsurahamanus*, displaying 80.57% and 81.14% identity values, respectively; these sequences are related to ORF21 more than to any other ORF21 homologous protein (Supplementary Figure S2). Moreover, the clustering of ORF21 and related homologous proteins from CA producers is robust with a branch support of 100% (Supplementary Figure S2). Additionally, the ORF21 protein was phylogenetically grouped with SigL proteins from the genus Streptomyces, as shown in Supplementary Figure S3.

**Figure 6:**
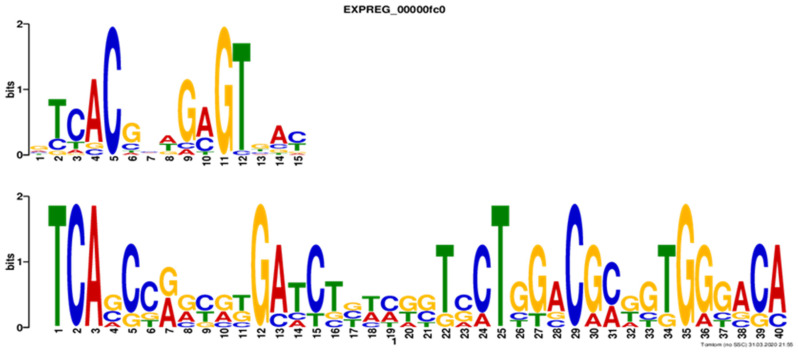
At the top, putative motif found in TOMTOM (*p*-value: 5.4 × 10^−4^ against a collection of bacterial transcription factors. TOMTOM showed that the query motif resembles the binding motif for transcription factor BldD. At the bottom, putative binding motif logo identified by MEME suite (E-value: 2.8 × 10^−6^) in the promoter region of the *claR* gene showing affected transcription in *S. clavuligerus*/pIORF21.

Figure 6 shows that, out of the six promoter regions analyzed, using MEME, the promoter region corresponding to *claR* from *S. clavuligerus*, *S. jumonjinensis*, and *S. katsurahamanus*, as well as *adpA*, *ccaR*, and *gcas* genes from *S. clavuligerus*, harbored the putative motif observed in Figure 6. The motif, analyzed using TOMTOM, was found to share homology to the binding motif for transcription factor BldD, a known regulatory motif from *S. coelicolor* A3(2) (Figure 6).

## 4. Discussion

Secondary metabolite production and morphological differentiation in bacteria are controlled by signal molecules that bind to cellular receptors and trigger intracellular regulatory cascades [30]. In this process, genes are activated and repressed to adapt the bacterial metabolism to different conditions, e.g., by producing antibiotics to compete with other microorganisms under limited nutrient conditions. RNA polymerase (RNAP) joins the promoter region by sigma factors, directing the expression of specific genes [31]. One of the most important and diverse groups of sigma factors are the extracytoplasmic function σ factors (ECFs) [32]. ECFs coordinate transcriptional responses to extracellular signals; yet, its role and mechanisms of regulation are largely unknown [30]. According to the ECF hub, an open access data repository for ECF classification, the putative SigL sigma factor (Supplementary Figure S3) encoded by *orf21* belongs to the actinobacterial ECF17 group [33]. The proteins present in the ECF17 group might be regulated by anti-σ factors [33]. Generally, a sigma factor is co-transcribed with a transmembrane anti-σ factor, with an extracytoplasmic sensory domain and intracellular domain [33]. Upon interaction with an extracytoplasmic signal (e.g., a protein or small molecule), a typical bacterial ECF factor is released and is free to bind to core RNAP and activate its regulon [34]. Thus, ORF21 would act at the cell membrane as a sensory protein that connects CA production with the environment.

The GSPG defined medium was chosen to evaluate the regulatory effect of ORF21 on CA biosynthesis due to its low levels of CA production, high salt concentration, limited amino acid content, as well as its known tendency to induce ammonium accumulation (Supplementary Figure S1) [11,21]. All these nutritional stress conditions and environmental imbalances in GSPG could affect the molecular mechanisms by which ORF21 step on CA regulation, substrate consumption, and morphological differentiation, compared to its regulatory role under nutrient rich conditions. In GSPG, the overexpression of *orf21* in *S. clavuligerus* increased the production of CA by 2.6-fold with a higher CA production per gram of substrate (Table 1). During cultivation, glycerol is used as the primary metabolic precursor of D-glyceraldehyde-3-phosphate (G3P), and as carbon source for maintaining cell metabolism [20]. In GSPG, *S. clavuligerus*/pIORF21 was more efficient than the control strain for redirecting carbon metabolic fluxes towards secondary metabolism rather than growth (Y_P/X_: 3,44 g·g^−1^) (Table 1). Conversely, ISP offers a nutrient rich condition under which high CA production is expected to be obtained; this is attributed to the use of a complex source of nitrogen that provides significant concentrations of free amino acids. In ISP, CA production by *S. clavuligerus*/pIORF21 was 55% lower compared to the control strain (see Figure 3b). In this case, low CA production was obtained per gram of biomass (Y_P/X_: 8,9 g·g^−1^) (Table 1). Hence, under these conditions, *S. clavuligerus*/pIORF21 apparently favors biomass production and primary metabolism related activities. An increase in CA production might be explained by substantial cell growth. In this study, we did not observe such a correlation between biomass and CA production (see Figure 4). Consequently, the diminished CA production was caused by the overexpression of *orf21* and its relationship with culture medium composition.

Unlike GSPG or the culture medium (SA) used by Jnawali et al. to produce CA, ISP offers, as the unique carbon source, a high content of glycerol (30 g/L), which is the carbon source preferentially used by *S. clavuligerus* to produce CA [35]. Therefore, a high concentration of glycerol and amino acids in ISP might lead to a metabolic scenario under which ORF21 favors primary metabolism in *S. clavuligerus*, as a strategy to manage metabolic resources. Conversely, in batch cultures employing glycerol and starch, as in the case of SA medium, the production of CA is diminished and the biosynthesis of cephamycin C prevails [35]. Likewise, the use of a mixture of sucrose and glycerol in a diauxic growth can repress CA production, since *S. clavuligerus* is unable to metabolize sucrose [36]. Particularly, GSPG and SA negatively affect CA production compared to ISP medium which favors secondary metabolism and therefore CA production in a process presumably mediated by ORF21.

**Figure 7:**
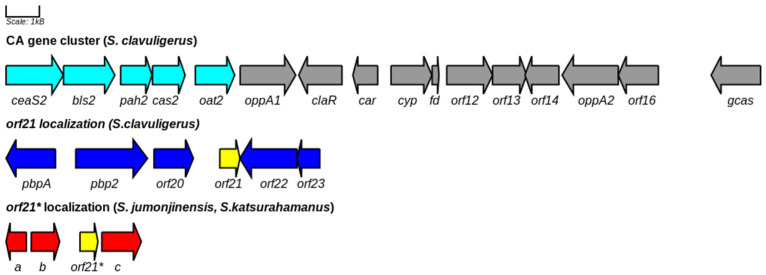
CA gene cluster organization in *S. clavuligerus* ATCC27064. Early genes in cyan and late genes in gray. *orf21* localization downstream of CA gene cluster was included for *S. clavuligerus* ATCC27064. In the bottom, the *orf21* homologous gene (orf21*), located far from the CA gene cluster in *S. katsurahamanus* and *S. jumonjinensis*, was included. *S. katsurahamanus* and *S. jumonjinensis*, share the same synteny for orf21* and neighboring genes. a: gene that encodes for response regulator transcription factor; b: gene that encodes for histidine kinase; and c: gene that encodes for a hypothetical protein.

*orf21* is located downstream of the CA gene cluster and upstream of *orf22* and *orf23* regulatory genes in *S. clavuligerus* ATCC 27,064 (Figure 7) [12]. In *S. clavuligerus* F613-1, the paired genes *cagS* and *cagR*, which are annotated, respectively, as *orf22* and *orf23* in *S. clavuligerus* ATCC 27,064, encode a bacterial two-component regulatory system, involved in fatty acid degradation, G3P and arginine metabolism [37]. Two-component regulatory systems constitute a family of proteins that mediate adaptation to changing environments by modifying the phosphorylated state of a pair of proteins: a sensor histidine kinase and a response regulator. Similarly, *orf21* may be part of an alternative mechanism that regulates carbon metabolism with CA biosynthesis in response to an environmental stimulus. To gain further insights about ORF21 incidence in primary metabolism, we analyzed the rate of glycerol uptake as an indirect measure of G3P direct primary metabolic precursor of CA. We found that overexpression of *orf21* reduced glycerol consumption rate in both the GSPG and ISP media (see Table 1).

In the wild-type strain of *S. clavuligerus*, about 20% of G3P is used as a precursor for CA biosynthesis and gluconeogenesis. The remaining 80% enters the glycolytic pathway through the GAPDH encoded by the *gap1* gene [38]. We suspect *orf21* overexpression would play a role in the transcription rate of genes related to the uptake and/or transport of glycerol into the cell, given the decline in glycerol consumption by *S. clavuligerus*/pIORF21 (see Figure 4) and considering that ECF factors such as ORF21 often control functions associated with cell surface or transport [30]. Although we did not evaluate the expression of genes related to glycerol metabolism in *S. clavuligerus*, transcriptional studies previously obtained in our laboratory (data not shown) suggest that *gap1* gene expression in ISP increased around 12-fold in *S. clavuligerus*/pIORF21 compared to the wild-type strain (Supplementary Figure S4). However, the presumption about the regulatory role of *orf21* on *gap1* must be experimentally evaluated using *S. clavuligerus*/pIB139 as control, in both ISP and GSPG.

To evaluate the effect of *orf21* overexpression on genes associated with CA production in GSPG, the transcriptional levels of well-known CA related genes were evaluated. We found no differences in amplification of *ccaR* transcript (Figure 5) between the evaluated strains. Nonetheless, Jnawali (2010) reported that ORF21 induces CA production by activating some genes (*ccaR*, *cas2*, and *ceas2*) involved in the early steps of the CA metabolic pathway, at 60 h of culture in SA medium [13]. The author suggests that ORF21 plays a fundamental role in the transcription initiation of many genes commonly expressed in the exponential growth phase. In the current study, RNA samples were taken at the stationary growth phase (96 h), which is associated with morphological differentiation and nutritional stress [39]. During this time, ORF21 might not only affect the early steps of CA formation, as mentioned by Jnawali, but also the late steps.

To assess the relationship between *orf21* and CA late steps, we studied the transcriptional levels of the *adpA* gene for the conditions assessed in this work. *adpA* encodes for the AdpA protein, a pleiotropic regulator which acts positively on the transcription of early and late genes of CA biosynthesis, as well as in biochemical and morphological differentiation of *S. clavuligerus* [11,22]. We found that *adpA* was slightly overexpressed (see Figure 5) in GSPG, which concurs with an improvement in CA concentration in GSPG (Figure 3a). Likewise, it was observed that *S. clavuligerus*/pIORF21 had a greater production of aerial mycelium (after 5–8 days) in contrast to the control strain (see Figure 2a,b). A greater production of aerial mycelium by *S. clavuligerus*/pIORF21 in GYM and MYM medium might be associated with an increase in the expression levels of *adpA* [10,28]. Similarly, we evaluated the transcriptional levels of the synthetic late gene *gcas*; we observed that *gcas* was partially overexpressed (see Figure 5). An increase in *gcas* overexpression would promote carbon flux towards CA biosynthesis, since the enzyme N-glycyl-clavaminic acid synthase controls the branch point between CA and the (3S, 5S)-clavams [40]. In addition, the enhancement in *gcas* expression agrees with the transcription rate of *adpA* [10]. Although, in the present study, an increase in the expression of the *ccaR* and *claR* genes was not observed, the slight improvement in the relative expression of *gcas* and *adpA* genes could have contributed to improve CA production in GSPG (see Figure 3a).

Furthermore, the transcription rate of some genes related to CA biosynthesis was evaluated to gain insights that might explain why ORF21 promotes a decrease in CA production in ISP. As previously mentioned, *S. clavuligerus*/pIORF21 grown in ISP favored primary metabolism (Table 1). Our results suggest that *orf21* overexpression indirectly decreases the transcriptional level of *claR* (see Figure 5). Martinez-Burgo et al. (2015) showed that a Δ*claR* mutant did not produce CA and exhibited a poor expression of the genes involved in the late steps of the CA pathway [33]. Consequently, the decrease in the transcriptional level of *claR* may possibly clarify the low CA titers obtained in this work, while using ISP (see Figure 3b).

**Figure 8:**
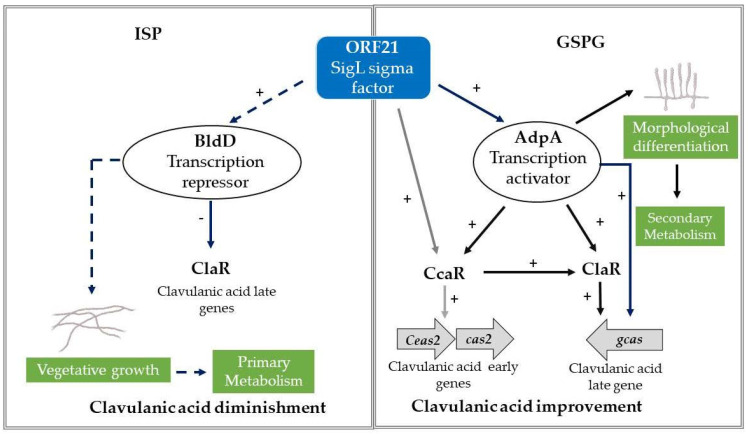
New insights about the regulatory character of ORF21 on the expression of some genes related to clavulanic acid biosynthesis (solid blue lines). Hypothetical mechanism of ORF21 acting on *claR* by *S. clavuligerus* in ISP medium (dotted blue line). Mechanism reported by Lopez-Garcia et al., 2010 (black lines) and Jnawali et al., 2011 (gray lines) [10,13].

Apart from *S. clavuligerus*, *S. jumonjinensis*, and *S. katsurahamanus* are the only species known to produce CA along with cephamycin C [1]. AbuSara et al. reported that the *orf21* gene is not present in *S. jumonjinensis* and *S. katsurahamanus* genomes, and therefore, it is not part of the core genes required for CA biosynthesis [1]. Although the genes that encode for WP_153520658.1 and WP_153484481.1 in *S. jumonjinensis* and *S. katsurahamanus* are located far from CA gene cluster and have a different synteny in regard to *S. clavuligerus* (see Figure 7), these proteins could be involved in the expression of genes related to CA biosynthesis through trans-regulatory mechanisms. Thus, the promoter regions of the *claR* gene of *S. jumonjinensis* and *S. katsurahamanus* were used as a support for in silico prediction of the theoretical DNA motif reported in Figure 6. The theoretically predicted DNA motif closely resembles the binding motif for the BldD (UnitProtKB: Q7AKQ8) transcription factor (Regulatory mode: 28% repression) of *S. coelicolor* (Figure 6). In *S. coelicolor*, BldD mainly acts as an overseer of vegetative growth and repressor of genes involved in morphological differentiation and/or secondary metabolism [41]. High-scoring copies of the BldD binding site have been found in the genomes of other bacteria containing a BldD homologue, suggesting that the role of BldD is conserved in sporulating actinomycetes [32]. Thereby, the presence of a BldD putative motif in the regulatory region of some CA biosynthetic genes could give some clues about the decrease in CA production obtained by *S. clavuligerus*/pIORF21 in ISP. Thus, we speculate the expression of SCLAV_0719 (*bldD*), for *S. clavuligerus* grown in ISP, may well be affected by *orf21* overexpression and, as a result, exert a negative regulatory influence on the transcriptional level of some genes related to CA production i.e., *claR*, *gcas*, and *adpA* (see Figure 5). On this subject, it might be reasonable to propose that ORF21 indirectly affects the transcription rate of *claR* by BldD (see Figure 8). Likewise, *bldD* expression could explain why primary metabolism prevails in *S. clavuligerus*/pIORF21 in ISP. Nevertheless, this inference must be further studied in works where the transcriptional level of the *bldD* gene in *S. clavuligerus*/pIORF21 would be evaluated.

## 5. Conclusions

Overall, the lack of knowledge and/or understanding of regulatory networks involved in *S. clavuligerus* metabolism constitutes a major bottleneck in CA overproduction. Therefore, the study of regulatory genes, e.g., *orf21*, provides further knowledge for overcoming these bottlenecks. Although *orf21* is not required for CA production, it plays an important role in morphological differentiation and in coordinating the transcription of some genes associated with CA biosynthesis during different stress responses. Likewise, its regulatory function interconnects CA biosynthesis with the primary metabolism of *S. clavuligerus*, allowing cell adaptation to different environmental conditions. With the present contribution, we established that ORF21 can affect the secondary metabolism of *S. clavuligerus* and improve CA production through the transcription initiation of genes involved in the late steps of CA pathway, such as *adpA* and *gcas* (Figure 8). AdpA is a regulator that positively affects CA metabolic pathway and morphological differentiation in *S. clavuligerus*/pIORF21. Therefore, an improvement in *adpA* expression might explain the relationship between ORF21 and the morphological differentiation of *S. clavuligerus*/pIORF21 in solid medium.

Conversely, under certain environmental conditions such as those presented in ISP, *orf21* overexpression negatively affects CA production, indirectly repressing the *claR* gene expression and once again demonstrating the regulatory character of ORF21 on CA late steps (Figure 8). Additionally, this contribution revealed that *orf21* expression affects primary metabolism of *S. clavuligerus* by regulating glycerol uptake. Just as the two-component system composed by *orf22* and *orf23* is fundamental for CA regulation, the sigma factor encoded by *orf21* is another environmental sensing system that *S. clavuligerus* must trigger for CA biosynthesis and cell adaptation. Consequently, the regulatory effect of ORF21 on CA production will depend exclusively on the conditions within which the cultivation is carried out. Hence, future RNA-seq studies will allow for a global exploration of the cellular metabolism of *S. clavuligerus*/pIORF21, thus enhancing our knowledge about CA biosynthesis and its regulation.

## Figures and Tables

**Table 1:** Kinetic parameters for CA biosynthesis from *S. clavuligerus* growing in GSPG and ISP media.

Culture Medium	Strain	Y_P/S_ (g·g^−1^)	Y_P/X_ (g·g^−1^)	Q_s_ (g·g^−1^·h^−1^)
GSPG	*S. clavuligerus*/pIORF21	0.0025	3.44	0.0063
	*S. clavuligerus*/pIB139	0.0005	1.4	0.0142
ISP	*S. clavuligerus*/pIORF21	0.2480	8.9	0.0003
	*S. clavuligerus*/pIB139	0.0234	17.6	0.0062

Y_P/S_: substrate to product yield; Y_P/X_: biomass to product yield. Qs: specific rate of substrate consumption.

## Data Availability

Not applicable.

## Supplementary Materials

The following are available online at https://www.mdpi.com/article/10.3390/bioengineering9020078/s1. Supplementary Material containing Table S1: Bacterial strains and plasmids used in this study. Table S2: Primers used in this study. Figure S1: Ammonium production during Streptomyces clavuligerus batch culture. Table S3: Normalized data for the relative gene expression. Figure S2: Phylogenetic tree of homologous proteins to Streptomyces clavuligerus ORF21 protein. Figure S3: Phylogenetic tree of some ECF sigma factors and ORF2. Figure S4: Effects of orf21 overexpression on the transcription of genes related to CA biosynthesis.
