# Supplementary material for: Environmental Factors Modulate the Role of orf21 Sigma Factor in Clavulanic Acid Production in Streptomyces Clavuligerus ATCC27064
Source: Bioengineering (Basel). 2022 Feb 16;9(2):78. doi: 10.3390/bioengineering9020078 (PMC8869649; doi:10.3390/bioengineering9020078)
Supplement: Supplementary file 1 [file bioengineering-09-00078-s001.zip › bioengineering-1557191-SI.pdf]

Environmental Factors Modulate the Role of *orf21* Sigma Factor in Clavulanic Acid Production in *Streptomyces Clavuligerus* ATCC27064

Luisa F. Patiño, Vanessa Aguirre-Hoyos, Laura I. Pinilla, León F. Toro and Rigoberto Ríos-Estepa*

Supplementary Table S1. Bacterial strains and plasmids used in this study

Strain/plasmid	Description	Source or reference
**Bacterial strains**		
S. clavuligerus ATCC 27064	Wild-type, CA andcephamycin producer.	Kindly provided bythe institute INBIOTEC at Leon University (Spain).
S. clavuligerus/pIORF21	*orf21* overexpressed in wild type *S. clavuligerus*.	This study
S. clavuligerus/pIB139	pIB139 without geneinsert integrated into *S. clavuligerus* ATCC27064 (control strain).	This study
Escherichia coli DH5	*F- 80lacZΔM15 Δ(lacZYA-argF) U169recA1 endA1 hsdR17 (r_k -,m_k +) phoA supE44 - thi-1 gyrA96 relA1*	General culturecollection. Bioprocessgroup at University ofAntioquia (Colombia).
Escherichia coli DSM11539 *(Migula 1985)*	GM48Sm^r (*thr leu thi lacY galK galT ara tonA tsx supE44), DeltalacproF1 traD36 proAB lacIQZDeltaM15*, lambda$^-$ Note: the strain has lost its dam and dcm markers.	DSMZ
**Plasmids**		
pTZ57R	*E. coli* general cloning vector, Amp^r.	Thermo Fisher Scientific, USA
pTZ57R/*orf21*	pTZ57R with *S.clavuligerus orf21* gene including *Xba*I-*Nde*Irecognition sites.	This study
pIB139	ϕC31 int and attP; aac(3)IV (apramycin); *erm*E* promoter.	Kindly provided bythe institute INBIOTEC at Leon University (Spain) [1].
pIORF21	pIB139 with *S. clavuligerus orf21* gene atits *Xba*I-*Nde*I recognition site.	This study

Supplementary Table S2. Primers used in this study.

Name	Primer sequence (5′-3′)	Description
orf21-1	FP:Ccccatatggtgattgccctgcgcgagagaac<br>RP:gggtctagaactaggcggtcacgccccgcttc	PCR amplification of *orf21*
aac(3)IV	FP: cagcttctcaaccttggggt<br>RP: atgatctgctctgcctgtgg	To confirm insertion of pIB139 in *S. clavuligerus.*
*ermE**	FP: atgctagtcgcggttgatcg	To confirm thetransformation of *S. clavuligerus* with pIORF21.
orf21-2	FP: gccggatcgtcatcaac<br>RP: cgacaggatctgactggt	Amplification of *orf21* by RT-qPCR
hrdB	FP: cgcggcatgctcttcct<br>RP: aggtggcgtacgtggagaac	Amplification of *hrdB* by RT-qPCR
gcas	FP: ggtcaactggagcctgtgta<br>RP: ccgcgaacttggcatagtc	Amplification of *gcas* by RT-qPCR
ccaR	FP: tcgcggactccatcgacctctt<br>RP: ggcgggccccttccacag	Amplification of *ccaR* by RT-qPCR
claR	FP: cgggcggcggttctt<br>RP: tcgtcgagcaggggttcc	Amplification of *claR* by RT-qPCR
adpA	FP: caggtctttaccagaggaaatc<br>RP: cgaactgctcgtgaagatg	Amplification of *adpA* by RT-qPCR
SCLAV_4359	FP: gtcggcgagaagatcgacat<br>RP: tgttgatgtagccggtgtcc	Amplification of SCLAV_4359 by RT-qPCR

Supplementary Table S3. Normalized data for the relative gene expression

Culture medium	Relative expression	*gcas*	*claR*	*adpA*
GSPG	$2^{(-\Delta\Delta C_t)}$	1.7	0.9	1.9
	Log 2 [$2^{(-\Delta\Delta C_t)}$]	0.7	-0.2	0.9
ISP	[$2^{(-\Delta\Delta C_t)}$]	1.4	0.005	1.4
	Log 2 [$2^{(-\Delta\Delta C_t)}$]	0.5	-7.7	0.5

Supplementary Figure S1. Ammonium production during *Streptomyces clavuligerus* batch culture. (**a**) GSPG medium and (**b**) ISP medium. *S. clavuligerus*/ pIORF21(◆) and *S. clavuligerus*/pIB139 (■). Each value is the mean of three flask replicates (n=3). Errorbars indicate standard deviation values.

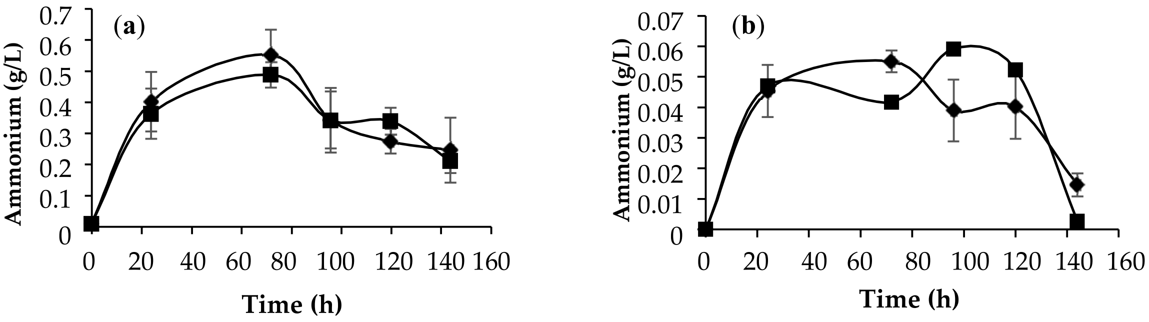

Supplementary Figure S2. Phylogenetic tree of homologous proteins to *Streptomycesclavuligerus* ORF21 protein. Homologous proteins were taken from NCBI BLAST (http:// www.ncbi.nlm.nih.gov/BLAST/ (accessed on 06 November 2021) search. In blue, the clustering for CA producer. The percentage of replicate trees in which the associated taxa clustered together in the bootstrap test (1.000.000 replicates) are shown next to the branches.

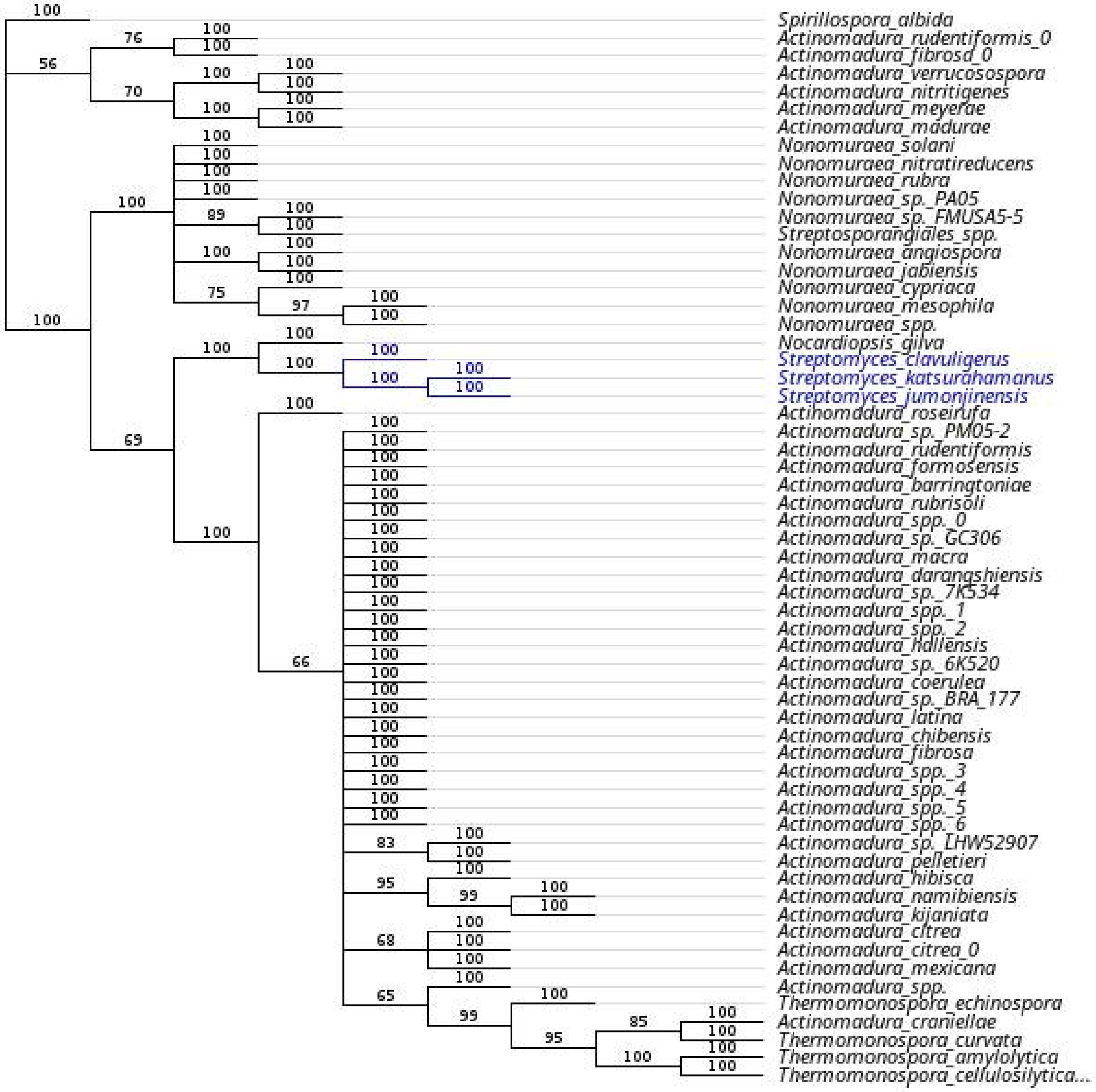

Supplementary Figure S3. Phylogenetic tree of some ECF sigma factors and ORF21. The GenBank accession numbers for the protein sequences used in the analysis are included with the names of each species. The clustering for SigL sigma factors is show in red. The percentage of replicate trees in which the associated taxa clustered together in the bootstrap test (1000 replicates) are shown next to the branches.

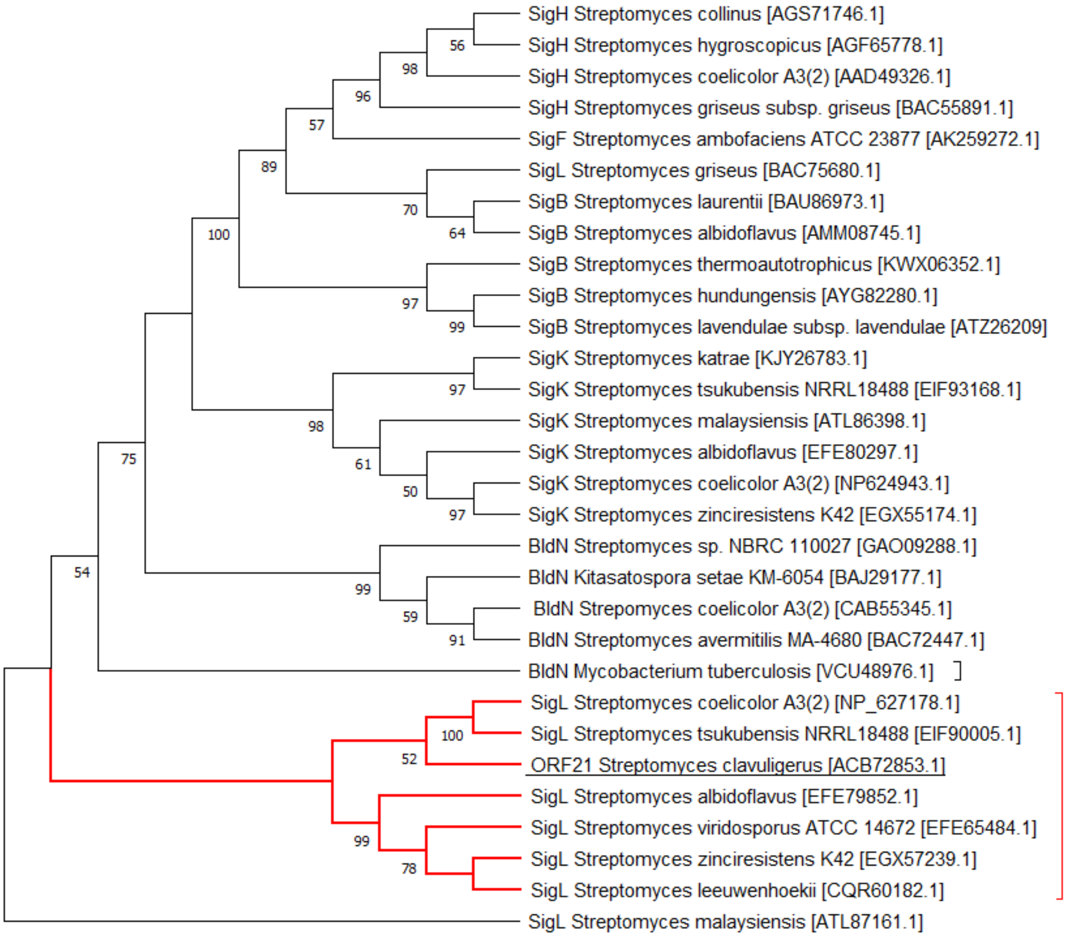

Supplementary Figure S4. Effects of *orf21* overexpression on the transcription of genes related to CA biosynthesis. RT-qPCR results for *S. clavuligerus*/pIORF21compared to *S. clavuligerus* wild-type strain in ISP. The RNA sample were taken at the peak of maximum CA production for both strains. The error bars indicate the standard error. Normalized data for the relative gene expression is shown in the table below.

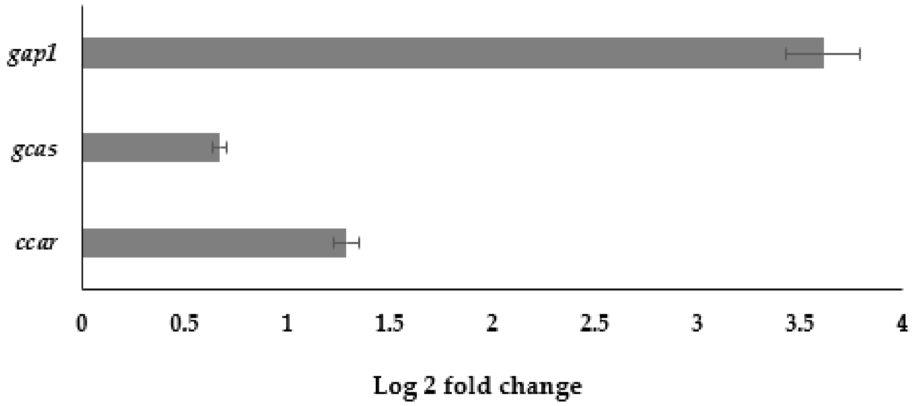

Culture medium	Relative expression	*gap1*	*gcas*	*ccar*
ISP	$2^{(-\Delta\Delta C_t)}$	12.3	1.6	2.4
	Log 2 [$2^{(-\Delta\Delta C_t)}$]	3.6	0.7	1.3

References

1. Wilkinson, C.J.; Hughes-Thomas, Z.A.; Martin, C.J.; Bohm, I., Mironenko, T.; Deacon, M.; Wheatcroft, M.; Wirtz, G.; Staunton, J.; Leadlay, P.F. Increasing the Efficiency of Heterologous Promoters in Actinomycetes. *J. Mol. Microbiol. Biotechnol.* **2002**, *4*, 417–426.
